# WGBSSuite: simulating whole-genome bisulphite sequencing data and benchmarking differential DNA methylation analysis tools

**DOI:** 10.1093/bioinformatics/btv114

**Published:** 2015-03-15

**Authors:** Owen J. L. Rackham, Petros Dellaportas, Enrico Petretto, Leonardo Bottolo

**Affiliations:** ^1^Program in Cardiovascular & Metabolic Disorders and Centre for Computational Biology, Duke-NUS Graduate Medical School, Singapore,; ^2^MRC Clinical Sciences Centre, Imperial College London, UK,; ^3^Department of Statistics, Athens University of Economics and Business, Greece and; ^4^Department of Mathematics, Imperial College London, UK

## Abstract

**Motivation**: As the number of studies looking at differences between DNA methylation increases, there is a growing demand to develop and benchmark statistical methods to analyse these data. To date no objective approach for the comparison of these methods has been developed and as such it remains difficult to assess which analysis tool is most appropriate for a given experiment. As a result, there is an unmet need for a DNA methylation data simulator that can accurately reproduce a wide range of experimental setups, and can be routinely used to compare the performance of different statistical models.

**Results**: We have developed WGBSSuite, a flexible stochastic simulation tool that generates single-base resolution DNA methylation data genome-wide. Several simulator parameters can be derived directly from real datasets provided by the user in order to mimic real case scenarios. Thus, it is possible to choose the most appropriate statistical analysis tool for a given simulated design. To show the usefulness of our simulator, we also report a benchmark of commonly used methods for differential methylation analysis.

**Availability and implementation**: WGBS code and documentation are available under GNU licence at http://www.wgbssuite.org.uk/

**Contact**: owen.rackham@imperial.ac.uk or l.bottolo@imperial.ac.uk

**Supplementary information:** Supplementary data are available at *Bioinformatics* online.

## 1 Introduction

The methylation of DNA is an important epigenetic modifier that is known to play a role in both development and disease. A growing number of studies are using whole-genome bisulphite sequencing (WGBS) to study the differences in methylation between samples and conditions (Barrero *et* *al.*, 2010). The number of statistical methods that have been developed to detect these differences from the data has grown considerably in recent years. However, it is difficult to judge the differences between these methods as they are not reliably benchmarked against each other.

Here, we present both a novel simulator of WGBS data and a benchmark of existing differential methylation techniques. Existing methods for simulating methylation data have been developed but these lack the complexity of real data or have been developed with reduced representation bisulphite sequencing in mind (Lacey *et* *al.*, 2013). By using two dependent Hidden Markov Models (HMM) (MacDonald and Zucchini, 1997), which can be fine tuned to approximate any WGBS dataset, we are able to simulate any genome-wide datasets taking into account spatial co-dependence, multiple methylation states (such as those in CpG islands or shores), read depth and number of replicate experiments. These simulations provide an ‘impartial’ data source through which existing and new statistical techniques can be compared, revealing that depending on the experimental setup and type of methylation differences that one wish to identify the choice of analysis may vary.

## 2 Materials and methods

Within the cell DNA methylation is a highly context dependent phenomenon typically being associated to stretches of DNA containing a Cytosine followed by a Guanine (so called CpGs). It is well documented (Barrero *et* *al.*, 2010) that the location of these CpG sites is not randomly distributed across a genome but rather these appear in dense clusters [referred to as CpG islands (Jones, 2012)] flanked by stretches of less CpG-dense DNA (referred to as CpG shores). The status of a CpG site (i.e. methylated or de-methylated) is also highly dependent on the methylation status of the surrounding CpGs. The methylation of CpGs that are close together in a stretch of DNA are much more likely to be correlated that those sites that are further apart (i.e. spatial co-dependence). The reviewer asked that this sentence be changed to the following: As a result, it is often the case that, in a reasonably homogeneous tissue sample, a CpG island will either be mostly methylated or de-methylated.

Detecting methylation status often takes advantage of bi-sulphite treatment of DNA. A reaction which results in un-methylated sites undergoing a conversion from a guanine base to uracil whilst no effect is felt at methylated sites. DNA sequencing following bisulphite treatment results in sets of reads that either map to a methylated or un-methylated versions of the genome. Depending on the depth of the sequencing there is a different number of reads covering each CpG site (i.e. reads coverage).

### 2.1 Simulation

**Fig. 1.**
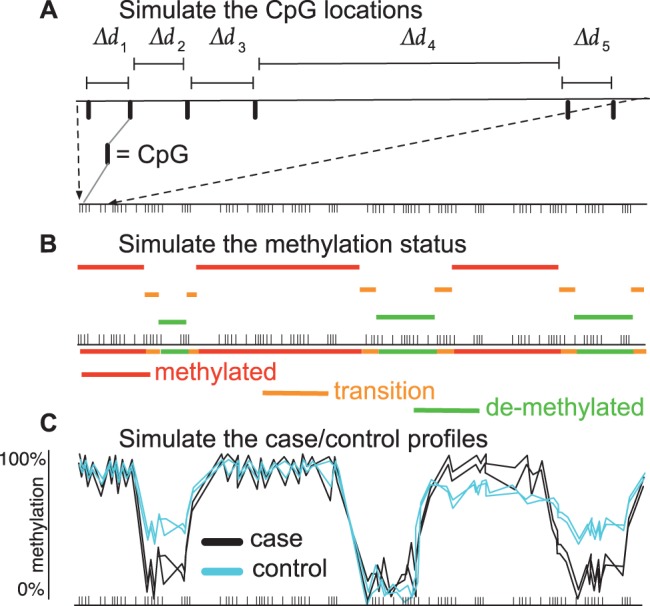
There are three stages (top-down) embedded in the DNA methylation data simulator. (**A)** Simulate the location of the CpGs using a homogenous discrete-state HMM. The first state emits short distances (CpG islands), the second state long distances (CpG deserts), and a third state that emits intermediate distances (CpG shores). (**B)** Simulate the methylation status at each CpG using a non-homogenous HMM, where the transitions between states are modulated by the distances of the CpG sites simulated in (A). (**C)** Each state assigned in (B) has a number of reads and methylated reads simulated from a Poisson and (truncated negative) binomial distribution, respectively

The model used to simulate single-base DNA methylation data is described in detail in Supplementary Information, outlined in Figure 1 and summarised as follows: (i) Simulate CpG locations: we use a homogenous discrete-state HMM with an exponential emission distribution to create CpG islands, shores and deserts. (ii) Simulation of methylation status at each CpG: we employ a non-homogenous discrete-state HMM so that CpGs that are close together are more likely to share the same state. (iii)Simulation of read depth at each CpG: a Poisson distribution is used to model the coverage. (iv) Simulation of methylated read counts: we use a binomial (or truncated negative binomial) distribution as the emission distribution of the non-homogenous discrete-state HMM.

### 2.2 Analysis

Since experimental design and sequencing technique can largely affect the resulting methylation dataset, we have also developed a tool to parameterize the simulation based on a real dataset provided by the user. Details are presented in the Supplementary Information, and the list of parameters that are automatically estimated are (i) Distance distribution: this defines the clustering of observations observed within CpG islands, shores and deserts.(ii) Probability of success: for each methylation status, this defines the probability of success in the (truncated negative) binomial distribution. (iii) Coverage distribution: this defines the expected number of trials in the Poisson distribution. (iv) Methylation difference between case and control groups: this defines the expected difference between the two groups in a differentially methylated region.

### 2.3 Benchmarking

**Fig. 2.**
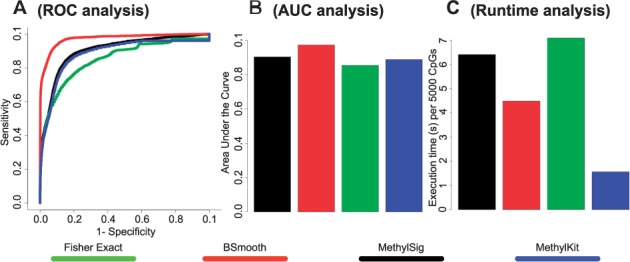
(**A**) A ROC analysis, (**B**) AUC analysis and runtime analysis of four different WGBS analysis techniques based on binomially simulated data

To show the usefulness of the DNA methylation data simulator, a benchmark is performed using several, commonly used and available packages for differential methylation analysis (described in detail in the Supplementary Information), including BSmooth (Hansen *et* *al.*, 2012), Methylseq (Li *et* *al.*, 2013), MethylKit (Akalin *et* *al.*, 2012) and the Fisher exact test. The test set can also be saved and used to incorporate other techniques not included as standard in the benchmarking. The result of the benchmark is a receiver operator characteristic (ROC) curve, the area under the curve (AUC) and a runtime plot, which can help the user to select the optimal method to use on their dataset (Fig. 2).

## 3 Discussion

WGBSSuite assists in the study of DNA methylation by supplying simulated DNA methylation datasets that are highly parameterisable based on real data provided by the user. The software has three sections which first allow a user to analyse their own experimental data (in order to find suitable parameters for the simulator) and then produce (and save) a simulated dataset of any size. Finally, the simulated datasets can be used to benchmark existing methods for differential methylation analysis, allowing the user to identify a suitable approach for their dataset. We acknowledge that this simulator does not deal with sample mixtures, an experimental setup that is common when methylation profiles are generated from complex heterogenous tissues (i.e. where the underlying signal is likely to originate from multiple cell-types). Future extensions will include a mixture distribution to model the probability of success of the non-homogenous discrete-state HMM emission distribution. This will allow the generation of single-base resolution DNA methylation data from complex heterogeneous tissues experiments such as those that arise from whole blood samples. Although this extra level of random variation can be easily included in our code, developing a simple estimator procedure for the parameters of the mixture distribution is not straightforward and it will be addressed in a future extension of WGBSSuite. Beyond providing an efficient tool to simulate WGBS datasets, our WGBSSuite will enable fast and efficient methods benchmarking, therefore facilitating the choice of the optimal analysis tool for differential methylation.

## Funding

This research was funded by Wellcome Trust (L.B), Medical Research Council UK (O.J.L.R., E.P.), European Union - European Social Fund (ESF) and Greek National Funds through the Operational Program ‘Education and Lifelong Learning’ of the National Strategic Reference Framework (NSRF) ARISTEIA-LIKEJUMPS (P.D.).

*Conflict of Interest*: none declared.

## Supplementary Material

Supplementary Data
