# Supplementary material for: WGBSSuite: simulating whole-genome bisulphite sequencing data and benchmarking differential DNA methylation analysis tools
Source: Bioinformatics. 2015 Mar 15;31(14):2371–3. doi: 10.1093/bioinformatics/btv114 (PMC4495289; doi:10.1093/bioinformatics/btv114)
Supplement: Supplementary Data [file supp_31_14_2371__index.html]

WGBSSuite: Simulating Whole Genome Bisulphite Sequencing data and benchmarking differential DNA methylation analysis tools — WGBSSuite: simulating whole-genome bisulphite sequencing data and benchmarking differential DNA methylation analysis tools — WGBSSuite: simulating whole-genome bisulphite sequencing data and benchmarking differential DNA methylation analysis tools — Supplementary Data 

# WGBSSuite: simulating whole-genome bisulphite sequencing data and benchmarking differential DNA methylation analysis tools

## Supplementary Data

files

**Files in this Data Supplement:**

- Supplementary Data - pdf file
