# Supplementary material for: WGBSSuite: simulating whole-genome bisulphite sequencing data and benchmarking differential DNA methylation analysis tools
Source: Bioinformatics. 2015 Mar 15;31(14):2371–3. doi: 10.1093/bioinformatics/btv114 (PMC4495289; doi:10.1093/bioinformatics/btv114)
Supplement: Supplementary Data [file supp_btv114_WGBSSuite_Appendix.pdf]

WGBSSuite: Simulating Whole Genome Bisulphite Sequencing data and benchmarking differential methylation tools

Owen Rackham, Petros Dellaportas,
Enrico Petretto and Leonardo Bottolo

A.1 Simulation details

In the following we detail the simulation steps embedded in the DNA methylation data simulator.

S.1 Simulate CpG locations: for a given set of CpG sites (T), we generate their locations l_t by simulating the distances between them using a discrete-state homogenous Hidden Markov Model HMM (HMM) (MacDonald and Zucchini, 1997) with a CpG island state, a CpG desert state and a CpG shore state. The transition matrix Π defines the probability of moving between the CpG island, shore and desert states

$$\begin{array}{c} \\ \text{island} \\ \text{shore} \\ \text{desert} \end{array} \begin{array}{c} \begin{array}{ccc} \text{island} & \text{shore} & \text{desert} \end{array} \\ \begin{bmatrix} \pi_1 & 1-\pi_1 & 0 \\ (1-\pi_2)/2 & \pi_2 & (1-\pi_2)/2 \\ 0 & 1-\pi_3 & \pi_3 \end{bmatrix} \end{array}$$

Given the state of the Markov chain at the tth CpG site, $t = 1, \ldots, T$, the distance between CpG_t and CpG_{t+1} is drawn from an exponential emission distribution with a fixed rate r (rate = 1/mean) different for each state (r_{island}, r_{desert} and r_{shore}). In the CpG island state this rate favours short distances (default = 0.025), the desert state long distances (default = $10e^{-4}$) and the CpG shore state medium length distances (default = 0.05). The location of each CpG is constant for each replica in each (case and control) sample.

S.2 Simulate methylation status: using the locations generated in Step S.1, the methylation status of each CpG site is modelled. This is done

using a discrete-state non-homogenous HMM, where the four states are: methylated, de-methylated and two transit states (methylated $\rightarrow$ 1st transit $\rightarrow$ de-methylated; de-methylated $\rightarrow$ 2nd transit $\rightarrow$ methylated). The transition matrix $\Pi(t)$ of the non-homogenous HMM is modulated by the distance between two neighbouring CpGs. Specifically,

$$\begin{array}{l} \\ \text{methyl.} \\ \text{1st transit} \\ \text{de-methyl.} \\ \text{2nd transit} \end{array} \begin{array}{c} \begin{array}{cccc} \text{methyl.} & \text{1st transit} & \text{de-methyl.} & \text{2nd transit} \end{array} \\ \begin{bmatrix} \pi_1(t) & 1-\pi_1(t) & 0 & 0 \\ 0 & \pi_2(t) & 1-\pi_2(t) & 0 \\ 0 & 0 & \pi_3(t) & 1-\pi_3(t) \\ 1-\pi_4(t) & 0 & 0 & \pi_4(t) \end{bmatrix} \end{array}$$

where $\pi_s(t) = a_s \exp\{-b \log(\min(1, l_t - l_{t-1})\}$, $s = 1, \ldots, 4$. l_t is the location of the tth CpG site, $a_s \in [0, 1]$ is the baseline probability of transition whereas the coefficient b (default $= 1.895e^{-02}$) controls how much $\pi_s(t)$ depends on the distance between two consecutive CpGs. For instance, the probability of methylation state of CpG_t being the same as CpG_{t-1} is a function of the distance between CpG_t and CpG_{t-1}, with greater distances favouring a change in state. Thus, in a CpG island, where CpG density is high, the chance of changing the state is low. It turns out that, on the whole, simulated CpG islands have (nearly) constant methylation status. The methylation status is constant for each replica in each sample.

S.3 Simulation of the coverage: at each CpG site, the number of reads n_t is simulated using a Poisson distribution with mean λ (default = 50). Alternatively, at each CpG site, the coverage is simulated using a Poisson distribution with mean λ_a or λ_b depending on the probability of methylation at each CpG site.

S.4 Simulation of methylated, un-methylated and transit reads: at the tth CpG site, given the methylation status simulated in Step S.2 and the coverage generated in Step S.3, the number of methylated (or un-methylated or transit) reads is modelled using a binomial emission distribution with n_t trials and a probability p_s of success different for each state ($p_{\text{methylated}}$, $p_{\text{un-methylated}}$ and p_{transit}). For simplicity the two transit states have the same probability of success. When the negative binomial density is chosen as the emission distribution, the number of simulated reads is truncated to n_t. To add noise to the number of simulated methylated (or un-methylated or transit) reads, an error term is added the probability of success p_s at the CpG site

$$p_{ts} = \frac{\exp\{\log(p_s/(1-p_s)) + d_t\}}{1 + \exp\{\log(p_s/(1-p_s)) + d_t\}},$$

$t = 1, \ldots, T$, $s = 1, 2, 3$, where $d_t \sim \mathrm{N}(0, s_0)$ with the variance s_0 controlling the level of noise.

Finally, a "phase difference" is applied to the probability of success (default = ± 0.1). The phase is defined as CpG sites where both case and control samples share the same methylated status. This parameter controls the extent of the reads difference between the two samples at each CpG site in each replica. The CpG sites where a "phase difference" is applied are selected as follows: (i) we select at random with probability proportional to the total length of the simulated methylated, de-methylated and transit sequence one of the three states; (ii) from the selected state, we draw with uniform probability (without replacement) one of the simulated sequence; (iii) we repeat steps (i) and (ii) until the total length of the "phase difference" is no grater than 5% of the total length of the simulated region. This guarantees that the "phase difference" can randomly happen and it is a small fraction of the whole simulated region.

A.2 Simulation parameters

Since the experimental design and the sequencing technique can vastly effect the resulting DNA methylation dataset, we have also developed an automatic tool that parameterises the simulation based on real data. This is done by estimate some of the simulator parameters directly as follows:

P.1 Calculate distance distribution parameters: depending on the organism of study or the approach used to obtain the DNA methylation data, the empirical distribution of CpG's position can vary from one experiment to another. To set the emission distribution parameters (r_{island}, r_{desert} and r_{shore}) of the discrete-state homogenous HMM (Step S.1 of the simulation), three separate exponential distributions are fitted. The first is limited to distances between CpGs that are a maximum of 200bps, the second is fitted to all CpG distances between 200bps and 1000bps and the third is fitted to all remaining CpG distances.

P.2 Calculate the coverage distribution parameter: the empirical distribution of reads across a given dataset is calculated. The empirical mean

is used as the parameter λ of the Poisson distribution of the coverage (Step S.3 of the simulation). Alternatively the user can chose to split the empirical distribution depending on whether the CpG has above/below 0.5 probability of methylated reads. In each case the mean is taken and it parameterized the Poisson coverage distribution.

P.3 Calculate the probability of success for each methylation status: to calculate the probability of success in methylated, de-methylated and transit regions, we firstly estimate $\hat{p}_t$, the Maximum Likelihood Estimate of the probability of success (number methylated reads / number of reads) at each CpG site, and then construct the empirical distribution of the MLEs. The resulting distribution is usually bimodal with two peaks, one close to zero and one close to one. By splitting this empirical distribution at 0.5, the median of the $\hat{p}_t$'s in the interval $[0, 0.5)$ and $[0.5, 1]$ can be used to estimate the probability of success in the methylated and de-methylated states (Step S.4 of the simulation). Finally, the probability of success for the transit states (for simplicity the two transit states have the same probability of success) is set equal to average probability of the methylated and de-methylated states.

P.4 Calculate the difference parameter in the methylation distribution between case and control samples: to estimate what a reasonable "phase difference" between case and control might be, the distribution of the difference of the probability of successes $\hat{p}_t$ between the two samples is computed. The resulting distribution in nearly centred around zero and the standard deviation can be used to estimate the scale at which the "phase difference" can be adjusted, with a small standard deviation suggesting highly related sample types and vice versa.

A.3 Benchmarking

B.1 Run BSmooth: Bsmooth (Hansen *et al.*, 2012) is run using its default parameters and a fixed value for the absolute mean difference in methylation of 0.01 and varying the qcutoff parameter that control the FDR q-value threshold.

B.2 Run Methylseq: Methylseq (Li *et al.*, 2013) is also run using its default settings and with a fixed minimum cutoff for difference in methylation of 5 reads.

B.3 Run MethylKit: MethylKit (Akalin *et al.*, 2012) is run using its default settings.

B.4 Run Fisher exact test: Fisher exact test using the routine in Hansen *et al.* (2012).

The result of the benchmarking is a Receiver Operator Characteristic (ROC) curve which is used to show how the specificity and sensitivity of each technique changes. This information is summarized in the Area Under the Curve (AUC) graph which highlights the overall capability of each technique. A plot of runtime of each technique is also provided.

References

Akalin, A., Kormaksson, M., Li, S., Garrett-Bakelman, F. E., Figueroa, M. E., Melnick, A., and Mason, C. E. (2012). methylKit: a comprehensive R package for the analysis of genome-wide DNA methylation profiles. *Genome Biology*, **13**(10), R87.

Hansen, K. D., Langmead, B., and Irizarry, R. A. (2012). BSmooth: from whole genome bisulfite sequencing reads to differentially methylated regions. *Genome Biology*, **13**(10), R83.

Li, S., Garrett-Bakelman, F. E., Akalin, A., Zumbo, P., Levine, R., To, B. L., Lewis, I. D., Brown, A. L., D'Andrea, R. J., Melnick, A., and Mason, C. E. (2013). An optimized algorithm for detecting and annotating regional differential methylation. *BMC Bioinformatics*, **14 Suppl 5**, S10.

MacDonald, I. L. and Zucchini, W. (1997). *Hidden Markov and other models for discrete-valued time series*, volume 110. CRC Press.
